# Do Search Engine Helpline Notices Aid in Preventing Suicide? Analysis of Archival Data

**DOI:** 10.2196/12235

**Published:** 2019-03-26

**Authors:** Qijin Cheng, Elad Yom-Tov

**Affiliations:** 1 Department of Social Work The Chinese University of Hong Kong Hong Kong China (Hong Kong); 2 Department of Social Work and Social Administration The University of Hong Kong Hong Kong China (Hong Kong); 3 Microsoft Research Herzeliya Israel; 4 Faculty of Industrial Engineering and Management Technion Haifa Israel

**Keywords:** search engines, suicide

## Abstract

**Background:**

Search engines display helpline notices when people query for suicide-related information.

**Objective:**

In this study, we aimed to examine if these notices and other information displayed in response to suicide-related queries are correlated with subsequent searches for suicide prevention rather than harmful information.

**Methods:**

Anonymous suicide-related searches made on Bing and Google in the United States, the United Kingdom, Hong Kong, and Taiwan in a span of 10 months were extracted. Descriptive analyses and regression models were fit to the data to assess the correlation with observed behaviors.

**Results:**

Display of helpline notices was not associated with an observed change in the likelihood of or future suicide searches (*P*=.42). No statistically significant differences were observed in the likelihood of people making future suicide queries (both generally and specific types of suicide queries) when comparing search engines in locations that display helpline notices versus ones that do not. Pages with higher rank, being neutral to suicide, and those shown among more antisuicide pages were more likely to be clicked on. Having more antisuicide Web pages displayed was the only factor associated with further searches for suicide prevention information (hazard=1.18, *P*=.002).

**Conclusions:**

Helpline notices are not associated with harm. If they cause positive change in search behavior, it is small. This is possibly because of the variability in intent of users seeking suicide-related information. Nonetheless, helpline notice should be displayed, but more efforts should be made to improve the visibility and ranking of suicide prevention Web pages.

## Introduction

Suicide prevention has been recognized as a global imperative by the World Health Organization [1]. Systematic reviews of suicide prevention strategies found that restricting access, physically and cognitively, to means of suicide is crucial for suicide prevention [2,3]. With the rapid increase in internet usage globally, Web-based search engines have become a crucial gateway for people to access information on suicide methods or places to access the means for suicide [4-6]. However, search engines can also facilitate people to find helping resources or access to Web-based support [7-9].

Since 2010, search engine providers have been gradually adding a box with information of the contact details of a suicide helpline when suicide-related queries are made [10]. Hereafter, we will refer this intervention as *helpline notice* or *the notice*. It is hoped that the prominent placement of helpline notices can attract suicidal individuals to utilize intervention resources rather than to further look for harmful information. However, other than the special placement of helpline notices, search engine providers use an automated algorithm to rank search results to satisfy the user’s information need by matching a document to a user’s search query and preference [11]. In other words, even though a helpline notice is displayed at the top of a search result page, information about suicide methods might also be shown on the search results page when a person queries for, for example, *how to kill myself*. In a situation like this, will the user read the helpline notice, harmful information about suicide methods, or both?

Although examination of the information returned by search engines when searching suicide-related queries has been conducted in multiple countries [5,7,8,12-18] to the best of our knowledge, no research exists on whether helpline notices successfully and sufficiently direct people to read information on prevention rather than harmful information, such as information about suicide methods or encouragement to suicidal behavior.

In view of the research gap and social significance, this study was conducted with an aim to answer the following research questions:

1. To what extent is the display of helpline notices associated with search engine user’s clicks on different types of suicide-related information?
2. Beside the presence of helpline notices, what other search characteristics, such as search queries, search timing (ie, when a search was made), and display availability of different types of information predict whether or not users would click through different types of suicide-related information?
3. Would the presence of helpline notices and the presence of antisuicide information in the search results prevent people from further searching for prosuicide information? Would such presence facilitate further searching for suicide prevention information?

## Methods

### Study Regions

The United States (US), the United Kingdom (UK), Hong Kong (HK), and Taiwan (TW) were chosen as the study regions for 2 major reasons. First, both Google and Bing search engines are freely accessible in the 4 regions. At the time of data collection, Google provided helpline notices in all 4 regions, whereas Bing provided such notices only in the US and UK. Therefore, a comparative analysis can be performed stratifying regions and search engines. Second, US and UK can be viewed as representatives of the English-speaking cultures, whereas HK and TW are viewed as representatives of the Chinese-speaking cultures, which allows for a cross-cultural comparison. Mainland China, although population wise is more comparable with UK and US, was not chosen because it implements rather strict censorship on the internet, and Google search is blocked there.

Aside from the abovementioned differences, the 4 jurisdictions share commonalities that allow for a comparison: they all have very high internet penetration rates (87% to 95%) and relatively high freedom of speech, where suicide is not criminalized, and no specific legal statute regulates suicide-related information. The 4 jurisdictions report different patterns regarding suicide: the age-standardized suicide rate in the UK (6.2 per 100,000) is lower than the other 3, followed by HK (8.8 per 100,000), US (12.1 per 100,000), and TW (13.1 per 100,000). The latter 3 had comparable rates [1] of suicide. All the 4 jurisdictions have suicide prevention hotlines available 24/7 for free for nation-wide callers. The types of suicide-related information which can be accessed by search engines have been examined individually in each of the 4 regions [7,8,12,14] (see details in the Multimedia Appendix 1). An initial comparison found that people in HK might be exposed to less prosuicide information through Web-based search than the other 3 regions. We noted, however, that those studies did not use consistent assessment protocols.

Informed consent was waived by the IRB. This study was approved by the IRBs of The University of Hong Kong (for labeling of data) and by the Technion (for the offline analysis of archival data).

### Data Collection

We used data from 2 primary sources:

Dataset 1—All searches made on the Bing search engine by people in the 4 regions between November 2016 and August 2017 (a total of 10 months). For each user we collected an anonymous user identifier, the region from where the user made the search, time and date, the text of the query, and the pages shown to people in response to the query and which pages they clicked on.

Dataset 2—A sample of anonymized logs from consenting users of a widely distributed Web browser add-on toolbar associated with the Microsoft Internet Explorer Web browser. Only visits to search engines were logged from these data. For each user in these data, we collected an anonymous user identifier, the region from where the user made the search, time and date, and the text of the query. These data allow cross-search engine comparisons.

The data were filtered to include only suicide-related queries. These queries were identified in 2 ways: English-language queries were those that triggered a helpline notice by Bing during June 2017 and Chinese-language queries were those queries that triggered helpline notices by Google in HK and TW. We noted that, to the best of our knowledge, helpline notices are triggered for a predefined list of query terms.

Owing to the different population sizes and to obtain a large enough sample from all 4 regions from dataset 1, we focused on those users who made suicide queries from US and UK in June 2017, and between January and June 2017 for HK and TW. Data from outside these dates were used for analysis of searches by users before and after they made suicide searches. In dataset 2, we focused on users who made suicide queries during June 2017 and tested for prior and post searches using data from May 1, 2017, to August 31, 2017.

Suicide-related queries in this study can be grouped into 2 categories: (1) queries which are associated with suicide prevention resources (referred as *suicide prevention queries* hereafter, eg, *helpline*, *lifeline*, *national suicide*, *crisis*, *hotline*, *chat*, *Samaritans*, or the telephone numbers of well-known local suicide prevention hotlines) and (2) queries associated with suicidal thoughts, behaviors, or suicide methods (referred as *suicide method queries* hereafter, eg, *poison*, *pills*, *hanging*, or *pesticide* but excluding the irrelevant phrases such as *poison ivy*). For both these query types, the Chinese-language equivalents were also included.

During data collection, we noted that the number of suicide-related searches and the number of frequently displayed Web pages varied in the 4 regions. To make a balanced comparison, in each country we selected 100 Web pages most often displayed to people in response to suicide-related queries. As there were only 91 Web pages displayed 10 or more times in HK, all of the 91 pages were assessed.

### Content Coding

Following previous examinations on Web pages’ bias toward suicide [13-14], we classified the Web pages into 6 types: antisuicide (ie, about suicide prevention or discouraging suicide), prosuicide (ie, the main content is encouraging suicide, romanticizing suicide, or introducing detailed suicide methods), neutral (ie, factual information about suicide incidents or statistics without notable bias), mixed (ie, mixed prosuicide and antisuicide information in the same Web page), not a suicide site (using *suicide* as a metaphor or joke, eg, animal suicide and political suicide), or error (ie, the Web page or major content of the page cannot be found). One modification on the previous commonly used coding frame is that we split the code Neutral/Mixed to 2 codes Neutral and Mixed because we suspect the 2 types of contents may have different impacts on vulnerable individuals. Examples of the 6 types of Web pages can be found in the Multimedia Appendix 1.

The assessment was performed by 6 trained coders. The training was provided by the first author and included guided reading of a booklet of recommendations on how to responsibly represent suicide-related information in mass media and Web-based media [19], and group exercise with coding 20 sample Web pages together. After training, each region’s dataset was assigned to 2 coders to conduct the classification independently. The codes agreed by the both coders were assigned as the final classification results. In total, 87% to 95% of each region’s data received agreed coding in the first round. For the remaining disagreed items, a third coder (ie, the first author) joined in the 2 coders to discuss the items until arriving at a consensus.

### Data Analysis

Descriptive analysis was first conducted to summarize the proportion of different types of Web pages being displayed in the search results in each study region. To answer Q1 & Q2, we used both descriptive statistics of dataset 2 and a logistic regression model fit to the data from dataset 1, with whether a Web page was clicked as the dependent variable (DV). The independent variables (IVs) included 3 types: (1) characteristics of a specific search, including when and where the search was made, whether or not helpline notice was displayed in the search results, and fractions of displayed Web pages in each type (calculated as the number of a certain type of pages being displayed divided by the number of total displayed pages) in the search results; (2) characteristics of a specific Web page in the search results, including ranking of the Web page, and what bias toward suicide the Web page shows; and (3) characteristics of the same user’s previous search within 7 days, including the total number of search queries made in 7 days, and the average number of search queries made every day. The latter was included to represent the level of Web-based activity of individual users. A total of 20,077,272 observations were included in this analysis.

To answer Q3, a hazard model was fit to the data from dataset 1, with 2 DVs: whether in the next 7 days the same user searched for (1) suicide method queries or (2) suicide prevention queries. The IVs included fractions of each type of page being displayed, fractions of each type of page being clicked, whether or not the helpline notice is being displayed, and the terms used in initial search queries. As only people who were active for at least 7 consecutive days were eligible for this analysis, a total of 154,286 observations were included in this analysis.

## Results

**Figure 1:**
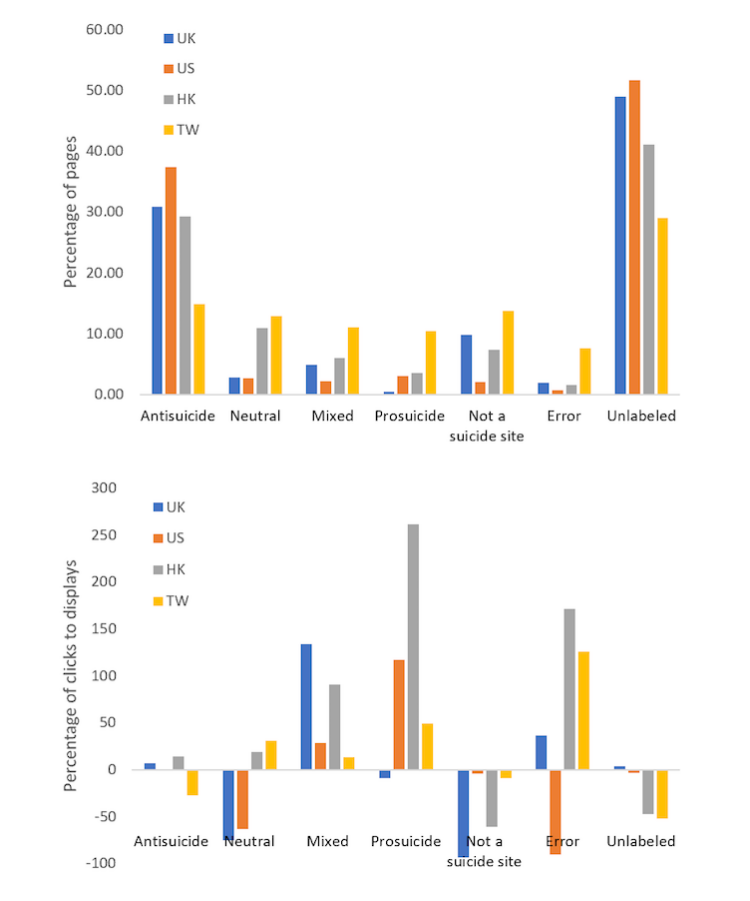
Characteristics of displayed and clicked Web pages in response to suicide-related search queries in the 4 study regions. Unlabeled pages are pages displayed fewer than 10 times in the study period. HK: Hong Kong; TW: Taiwan; UK: United Kingdom; US: United States of America.

Figure 1 shows the distribution of displayed pages in the 4 regions of Bing search in terms of their bias toward suicide. As the figure shows, the vast majority of pages frequently shown to users are antisuicide pages across the 4 regions. However, in TW the frequency of displayed Web pages contained a higher proportion of prosuicide (13.8%) but lower proportion of antisuicide information (15.0%) compared with the other 3.

Figure 1 also shows the click-to-display ratio by region and type of pages. The ratio is calculated as follows: (% clicks/%displays−1)×100. Despite a smaller proportion in frequently displayed pages, prosuicide and mixed pages were much more likely to be clicked in all but the UK. Conversely, although antisuicide pages were more likely to be displayed, their click-to-display ratio appeared to be low across the 4 regions and were even negative in TW. In addition, neutral pages were more likely to be clicked in HK and TW but not so in UK and US when being displayed. Keywords from queries associated with the highest probability of clicking on a specific page type are shown in the Multimedia Appendix 1.

An analysis of the association between clicks on helpline notices and other behaviors on the search page shows that 83.7% of people who clicked on the notice also clicked on other links compared with 91.5% of those who did not click on the helpline notice (χ^2^=59.3, n=49,950, *P*<.001). Clicks on helpline notices are not associated with a change in the likelihood of future suicide searches (40.1% when not clicked, 38.6% when they are, χ^2^=0.7, *P*=.42).

Table 1 shows the logistic regression model parameters for predicting whether a user would click on a page given its parameters and those of the user and their past search behaviors. As the table shows, helpline notice displays are not statistically significantly associated with a change in click behavior. A page with higher rank being neutral to suicide and having more antisuicide pages displayed in the same results list were more likely to be clicked on. That page’s probability to be clicked would be even higher when a search was made in later time of a day (as the slope is positive) and an early day of a week (negative slope, where days of the week are coded from Sunday (1) to Saturday (7)). In terms of regions, the click behavior in UK differed from that of the other regions. Previous levels of search activity did not show significant influence on one’s click behavior in the model.

**Table 1:** Parameters of the logistic regression model to predict clicks on a page. The United States has been used as the baseline region in the cross-region model, and neutral pages as the baseline for webpage types.

Parameters		Estimation (SE)	*P* value
**Search results characteristics**			
	Is helpline displayed	9.333 (34)	>.99
	Fraction of antisuicide displayed	0.817 (0.037)	<.001
	Fraction of neutral displayed	0.664 (0.109)	<.001
	Fraction of mixed displayed	−0.851 (0.072)	<.001
	Fraction of prosuicide displayed	−0.766 (0.053)	<.001
**Page characteristics**			
	Rank	0.205 (0.002)	<.001
	Antisuicide	−1.034 (0.033)	<.001
	Prosuicide	−1.073 (0.039)	<.001
	Mixed	−1.32 (0.038)	<.001
**Time**			
	Hour of the day	−0.006 (0.001)	<.001
	Day of the week	0.008 (0.003)	.003
**Region**			
	United Kingdom	−0.882 (0.015)	<.001
	Hong Kong	−10.026 (34)	>.99
	Taiwan	−10.1 (34)	>.99
**Previous search characteristics**			
	Number of previous queries	0.000 (0.000)	.46
	Previous queries per day	0.000 (0.000)	.004

Table 2 shows the hazard model parameters for predicting searches for suicide method queries in the next 7 days. As the table shows, people who were exposed to more suicide-related pages were associated with higher likelihood of further searching for suicide method queries. Among terms, initial search queries of *suicide* and *kill (myself)* were associated with higher risk to further search suicide methods, as opposed to *suicide hotline*. Some terms, however, were less expected in their association with increased or decreased hazards. For example, people who queried for *kill yourself* or *kill myself* were less likely to further search for suicide methods. The model for future suicide prevention queries found only one statistically significant variable—the fraction of antisuicide pages displayed (hazard=1.182, *P*=.002).

Using dataset 2, we compared user behaviors, stratified by region and search engine. Table 3 shows the probability that users will make future suicide-related queries, future suicide method queries, or future helpline queries, given their region and search engine (*P* values are calculated using the chi-square test). As shown in the table, no statistically significant difference can be found among the users of any of the 4 regions. The finding is consistent with the hazard model’s results.

**Table 2:** Hazard model parameters for predicting future suicide methods searches. The following are attributes with statistically significant results (*P*<.05).

Parameters	Hazard ratio	*P* value
Fraction of antisuicide displayed	1.0131	<.001
Fraction of neutral displayed	1.0811	<.001
Fraction of mixed displayed	1.0702	<.001
Fraction of prosuicide displayed	1.0428	.002
Number of previous suicide queries	1.0009	<.001
Term: *suicide*	1.4137	<.001
Term: *kill*	2.4258	<.001
Term: *suicide hotline*	0.8088	<.001
Term: *to kill*	1.2231	<.001
Term: *kill yourself*	0.5616	<.001
Term: *suicidal*	1.3115	<.001
Term: *kill myself*	0.5247	<.001

**Table 3:** The probability of further searching for overall suicide queries, suicide method queries, and suicide prevention queries by region and search engine. *P* values calculated using the chi-square test.

Country	All suicide queries			Suicide methods queries			Suicide prevention queries		
	Bing	Google	*P* value	Bing	Google	*P* value	Bing	Google	*P* value
United Kingdom	0.457	0.508	.27	0.009	0.008	.91	0.009	0.004	.50
United States	0.467	0.498	.20	0.008	0.014	.27	0.012	0.016	.46
Hong Kong	0.696	0.750	.67	0.261	0.357	.46	0.087	0.036	.44
Taiwan	0.801	0.755	.31	0.635	0.612	.66	0.000	0.005	.36

## Discussion

As Web-based search engines are commonly used by people as the gateway to information, it is imperative to examine how the information returned by search engines influences people’s behaviors for the purpose of suicide prevention. To the best of our knowledge, this study is the first to empirically examine the helpline notice intervention’s impacts, doing so across geographic regions, languages, and search engines.

Our results suggested that helpline notices do no harm, as it was neither associated with greater clicks on prosuicide pages nor with further search for suicide method information. However, the display of helpline notices was not associated with a higher observed likelihood of clicking on or further searching for suicide prevention information. Although a previous study found that the prominent placement of the helpline notice draws people’s attention [20], our results suggest that more views do not result in more click-throughs. This might be related to the fact that the helpline notices provide a helpline number, using which people can directly call the helpline without clicking on search results. Alternatively, people searching for suicide-related information may be doing so for a variety of reasons. There might be only a small group of users whose searching behaviors were affected by the display of helpline notice.

Compared with the display of the helpline notice, the types of Web pages displayed in the search results and the search queries people made appear to be more predictive of what people read and further search for. We posit that search queries may reflect the users’ suicide risk intention and psychological needs [21]. For example, those who searched for the suicide hotline might be willing to seek help and would less likely further search for suicide methods. However, it seems to be surprising that those who initially searched for *kill yourself* or *kill myself* were also less likely to later search for suicide methods. A potential explanation is that those people might be satisfied by the information they found in their initial search or have taken actions to harm themselves after the initial search, which reduced their likelihood of further searching.

More displays of antisuicide Web pages in the search results were found to be the only factor associated with further searching for suicide prevention information, after controlling for what search queries were used initially. The result suggests that, when a person is exposed to more antisuicide information, regardless of their initial mental status and needs, their interests in suicide prevention information might be stimulated or their information need might be unsatisfied, leading to additional queries. The finding might also be explained by the planned behaviors theory that people’s behavior is influenced by what they perceive as the social norm [22,23]. When more antisuicide information is displayed, it may create an impression that suicide prevention is a widely accepted norm and attract people to learn more about it.

People were exposed mostly to antisuicide pages in the results displayed by Bing. We note that, among the 4 study regions, people in TW saw more prosuicide and fewer antisuicide pages in their search results, and their antisuicide pages got the lowest click-to-display ratio. The findings are consistent with another study examining what suicide-related information was shown in the first 3-page search results in TW [14]. Coincidently, TW also reports the highest suicide rate among the 4. A previous study found that Web-based search for a new suicide method in TW was associated with more suicide incidents in the following days [24]. The special phenomenon in TW and its underlying reasons deserve more investigations. Anecdotal observations of the prosuicide pages reveal that many of them were related to a book— *The Complete Manual of Suicide* —which described suicide methods in details and rated each method by its level of painfulness, probability of failure, and other dimensions. By contrast, the promotion of this book only appeared once in HK search results. The book was originally published in Japan and soon translated into Chinese by a TW publisher and sold in both TW and HK. Although the book was later banned from sale in TW and HK, its content is still accessible on the Web and might have caused greater impact in TW, given TW’s close cultural relation with Japan.

Some limitations of the study should be noted. First, we only selected 4 regions for this study, which does not allow us to generalize our results to the entire world. In addition, the included search queries were those that triggered helpline notices. There might be other search queries used for finding suicide information that were absent from the list. Nonetheless, the list included hundreds of terms in English and Chinese, which should have covered the most frequently used ones. Another limitation is that we used clicking on and further searching for certain types of information to indicate behavioral change. Other scenarios such as calling a helpline or acting on suicidal intentions without further searching were not addressed by these measures. Finally, users may be issuing their queries from multiple devices, on multiple services, or from shared devices. This can cause our analysis of future and prior searches to be affected, mostly in a way which underestimates the likelihood of future (or prior) searches.

This study suggested that helpline notices were not associated with harmful outcomes such as clicking on more prosuicide search results or searching for more prosuicide information. Given that a previous study found that people’s attention is focused on these notices, it should thus be continued so as to raise public awareness that suicide is preventable and local prevention resources are available. Other than the helpline notice, having more antisuicide pages being displayed in Web-based search results and in higher ranking may facilitate people to access more suicide prevention information. Suicide prevention organizations and other public health institutes, especially those in TW, should increase the antisuicide information’s visibility and attractiveness. In addition, health policymakers should be aware of the potential impacts of Web-based search and initiate directives or guidelines for appropriate Web-based information dissemination.

## Appendix

Multimedia Appendix 1 1. Background info of the four study regions. 2. Examples of six types of webpages. 3. Terms associated with the highest probability of clicking on a specific page type. 4. Screenshots of help notices from Hong Kong and from the US.

## Abbreviations

DV: dependent variable
HK: Hong Kong
IVs: independent variables
TW: Taiwan
UK: United Kingdom
US: United States
