# Supplementary material for: Do Search Engine Helpline Notices Aid in Preventing Suicide? Analysis of Archival Data
Source: J Med Internet Res. 2019 Mar 26;21(3):e12235. doi: 10.2196/12235 (PMC6454333; doi:10.2196/12235)
Supplement: Multimedia Appendix 1 [file jmir_v21i3e12235_app1.pdf]

**Supplementary Table 1. Background info of the four study regions**

| | **Hong Kong** | **Taiwan** | **United States** | **United Kingdom** |
|---|---|---|---|---|
| Population (2017)[a] | 7,364,883 | 23,626,456 | 324,459,463 | 66,181,585 |
| Internet penetration rate (2017)[b] | 87% | 87.9% | 87.9% | 94.8% |
| Age-standardized Suicide rate, per 100,000 people (2012)[c] | 8.8 | 13.1 | 12.1 | 6.2 |
| Proportion of different types of suicide information shown in the first-three-page search results by suicide-related search queries[d] | Pro-suicide: 2.8% Anti-suicide: 39.5% Neutral/mixed: 45.2% Irrelevant: 8.4% Error: 4.1% | Pro-suicide: 16.3% Anti-suicide: 41.3% Neutral/mixed: 33.3% Irrelevant: 7.2% Error: 1.9% | Pro-suicide: 11.7% Anti-suicide: 34.9% Neutral/mixed: 29.2% Irrelevant: 16.1% Error: 8.1% | Pro-suicide: 23.1% Anti-suicide: 24.4% Neutral/mixed: 48.6% Irrelevant: 9.4% Error: 0.8% |

***Data Source**:*

[a]*United Nations (2017). 2017 Revision of World Population Prospects. URL:* https://esa.un.org/unpd/wpp/

[b]*Internet World Stats. Internet Users in the World – December 31, 2017.* https://www.internetworldstats.com/stats.htm

[c]*World Health Organization (2014). Preventing Suicide: A World Imperative. Full report can be downloaded from:* http://www.who.int/mental_health/suicide-prevention/world_report_2014/en/

[d]*US data from Recupero et al. (2008); UK data from Biddle et al. (2016); Hong Kong data from Cheng (2012); Taiwan data from Chen et al. (2017). Because Biddle et al. (2016) used different coding scheme, their results are recoded by renaming "all dedicated suicide sites" as "pro-suicide," combining "against suicide sites," "prevention*

*or support sites," and "academic or policy sites" as "anti-suicide," combining "all information sites," 'news reports of individual suicides," "chatrooms/blog sites focused on suicide methods," "chatrooms/blog sites discussing various general issues relating to suicide," "miscellaneous sites reporting on topical issues relating to suicide," and "collections of images" as "neutral/mixed," combining "hit not relevant to search term" and "completely joking information" as "Irrelevant," and renaming "page not available" or "error."*

**Supplementary Table 2. Examples of six types of webpages.**

| | Hong Kong | Taiwan | United States | United Kingdom |
|---|---|---|---|---|
| Pro-suicide (i.e. the main content is encouraging suicide, romanticizing suicide, or introducing detailed suicide method) | http://www.ed2000.com/ShowFile.asp?FileID=173382 (promoting a book describing suicide methods in details- "The Complete Manual of Suicide") | https://sites.google.com/site/moonofmagick2/%E4%B8%8A%E5%90%8A%E5%8E%9F%E7%90%86%E5%8F%8A%E4%BD%8D%E7%BD%AE%E5%8F%83%E8%80%83 (detailed information about how to kill oneself by hanging) | http://lostallhope.com/suicide-methods | http://lostallhope.com/suicide-methods |
| Anti-suicide (i.e. about suicide prevention or discouraging suicide) | http://www.sbhk.org.hk/ (A local NGO for suicide prevention) | https://www.jtf.org.tw/suicide_prevention/ (A local NGO for suicide prevention) | https://suicidepreventionlifeline.org/ | https://www.nhs.uk/conditions/suicide/ |

| | Hong Kong | Taiwan | United States | United Kingdom |
|---|---|---|---|---|
| Neutral (i.e. factual information about suicide incidents or statistics without notable bias) | https://zh.wikipedia.org/zh-hk/%e5%90%84%e5%9b%bd%e8%87%aa%e6%9d%80%e7%8e%87%e5%88%97%e8%a1%a8 (wiki page about suicide rates in different countries) | http://tspc.tw/tspc/portal/howdo/index.jsp?sno=81 (wiki page about suicide) | https://en.wikipedia.org/wiki/suicide | https://en.wikipedia.org/wiki/suicide |
| Mixed (i.e. mixed pro-suicide and anti-suicide information in the same webpage) | http://www.failforum.net/forum/viewthread.php?tid=378026&extra=page=76&filter=0&orderby=dateline&page=1 | http://wotupset.eu5.org/tsl/_1308/1376142994232.htm (an online forum thread mixed comments supporting and criticizing "The | https://answers.psychcentral.com/story.php?title=i-want-to-die-what-is-the-easiest-way-to-do-that | http://www.insidermonkey.com/blog/7-easiest-painless-ways-of-killing-yourselves-quickest-360388/ |

| | Hong Kong | Taiwan | United States | United Kingdom |
|---|---|---|---|---|
| | (an online forum thread mixed comments encouraging and discouraging a person's suicidal thoughts) | Complete Manual of Suicide") | | |
| Not a suicide site (using "suicide" as a metaphor or joke. E.g. animal suicide, political suicide, etc.) | http://news.4399.com/wdshijie/ (a gaming website) | https://www.gq.com.tw/entertainment/movie/content-21717.html (about the movie "Suicide Squad") | http://deathclock.com | http://www.cracked.com/humor-history.html |
| Error (i.e. the webpage or major content of the page cannot be found) | http://www.miketso.com/unlucky_house/nt/yuenlong/tin_shui_wai/index.html | https://www.niusnews.com/=p2ue6j08 | https://answers.yahoo.com/question/index?qid=20080722234105aajvsu3 | http://uncyclopedia.wikia.com/wiki/howto:commit_suicide |

**Supplementary Table 3: Terms associated with the highest probability of clicking on a specific page type**

| **Page type** | **Terms** |
| --- | --- |
| Pro-suicide | best methods, by pills, common, common method, common suicide |
| Anti-suicide | am depressed, and suicidal, anonymous, anonymous suicide, chat line |
| Neutral | articles, hanging, numbers, org, rates |
| Mixed | 10 ways, com, committing, committing suicide, common suicide |

## Supplementary Table 4: Screenshots of help notices from Hong Kong and from the US

<table>
<tr><td>HK, Google</td><td>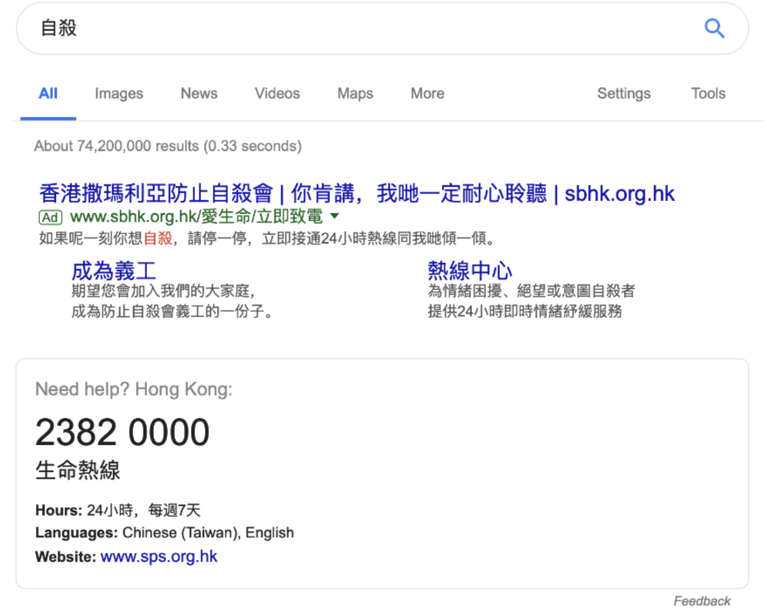
</td></tr>
<tr><td>US, Google</td><td>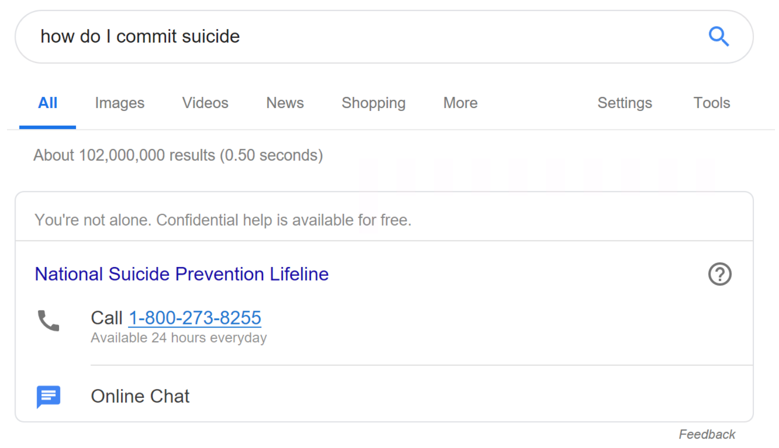
</td></tr>
<tr><td>US, Bing</td><td>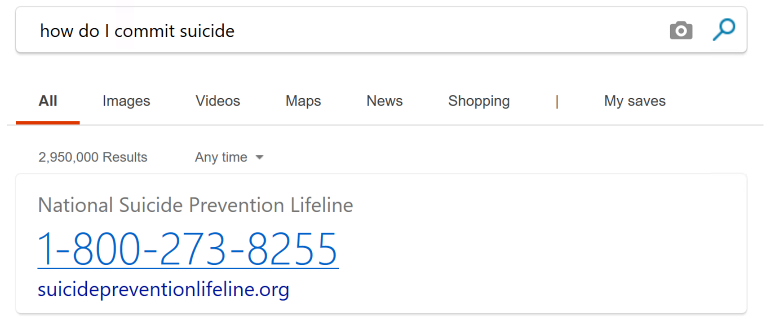
</td></tr>
</table>
